# Response to: A paleoecological context to assess the development of oak forest in Colombia: A comment on Zorrilla‐Azcué, S., González‐Rodríguez, A., Oyama, K., González, M.A. & Rodríguez‐Correa, H., The DNA history of a lonely oak: *Quercus humboldtii* phylogeography in the Colombian Andes. Ecology and Evolution 2021, doi:10.1002/ece3.7529

**DOI:** 10.1002/ece3.9271

**Published:** 2022-09-12

**Authors:** Sofía Zorrilla‐Azcué, Antonio González‐Rodríguez, Ken Oyama, Mailyn A. González, Hernando Rodríguez‐Correa

**Affiliations:** ^1^ Universidad Nacional Autónoma de México Morelia Mexico; ^2^ Posgrado en Ciencias Biológicas Universidad Nacional Autónoma de México, Unidad de Posgrado, Circuito de Posgrados, Ciudad Universitaria Ciudad de México Mexico; ^3^ Universidad Nacional Autónoma de México, IIES Morelia Mexico; ^4^ Laboratorio de Genética de la Conservación Instituto de Investigación de Recursos Biológicos Alexander von Humboldt Bogotá Colombia

## Abstract

In this response, we address comments and clarify the rationale behind the choice of hypotheses aimed to describe the *Quercus humboldtii* phylogeography in the Colombian Andes. Finally, we explain our disagreement with the conclusions of a previous critique, since these are not necessarily adequate under the implemented population genetics approach.

In a previous contribution to this journal, we submitted a paper on the phylogeographical history of the oak species *Quercus humboldtii* (Zorrilla‐Azcué et al., 2021). Using chloroplast and nuclear microsatellites, we characterized the patterns of genetic structure and diversity and proposed a hypothesis which aimed to understand possible responses of *Quercus humboldtii* to the environmental changes during glacial and interglacial cycles in the Andean Montane Forest. Shortly after, a comment by Hooghiemstra et al. (2022) was released in which they disagreed with some interpretations of our results in light of the paleoecological knowledge they have developed. They synthesized the current paleoecological understanding of the vegetation changes that occurred in the Northern Andes during the Pleistocene and proposed alternative interpretations to the genetic results.

Firstly, we appreciate the comments made by Hooghiemstra et al. (2022) to our recently published study (Zorrilla‐Azcué et al., 2021). We entirely agree with the fact that the integration of information from several scientific disciplines such as genetics, environment, geology and paleoecology is a key factor to better understand the complex evolutionary history of species and ecosystems, such as the Andean montane forests. In this response, we present several comments to attend to the main concerns of Hooghiemstra et al. (2022) regarding the interpretation of our results, as well as the hypothesis tested from a genetic approach.

One important point of agreement by both research groups is that, due to the topography of the Northern Andes and the altitudinal range *Q. humboldtii* occupied, it is likely that there was general connectivity among forest patches at a large scale. This means a high possibility of frequent gene flow reflected in low genetic structure and an effective population size large enough to allow the accumulation of genetic diversity.

This message was, in essence, what we were trying to establish in our discussion about *Q. humboldtii*'s genetic structure. As a complementary argument, we looked to establish a precedence with other phylogeographic studies of the Northern Andes montane forests, like Sanín et al. (2017) paper on *Ceroxylon* species, in which there was evidence of gene flow among geographic features that could have acted as barriers (like the low valleys between the main cordilleras). In their comment, Hooghiemstra et al. (2022) criticized our example by arguing that we were implying the lack of altitudinal descent of Ceroxylon populations during climate fluctuations.

However, the authors' formulation that “…the persistence of Ceroxylon populations is related to the relatively stable temperature and humidity conditions in Colombia during the last 350 ka…” is misleading. The high‐resolution temperature record from the Northern Andes (Bogotá‐A et al., 2016; Groot et al., 2011, 2013) shows substantial changes in temperature at millennium time‐scales and larger temperature changes superimposed parallel to changes in climatic humidity. Populations of the palm Ceroxylon, currently reaching up to 2800 m elevation, shifted downslope during colder (glacial) conditions as did all arboreal taxa of the UMF. In this way Ceroxylon continued to occur in “similar” temperature conditions as today (called “climate tracking”) (Hooghiemstra et al., 2022, page 3, 1st paragraph)

However, we disagree with this concern. In the original cited paper by (Sanín et al., 2017), they explain that the pattern of higher genetic diversity at the Colombian cordilleras might be due to demographic stability and increased immigration rates. One of the mentioned explanations is that:

… the cordilleras of Colombia have had relatively stable, moist, climatic conditions since 350,000 years before present.… Several periods of cooling, … are considered to have caused a general descent in the elevation of Andean forest of ca. 500 m (Veer & Hooghiemstra, 2000), that did not compromise their persistence. (Sanín et al., 2017, page 296, “Phylogeographical Patterns” 2nd paragraph)

And, this same idea was explained in our paper:

(Sanín et al., 2017) mentioned that even during glacial periods, it is likely that the altitudinal descent of the vegetation belt did not compromise the persistence and connectivity of Ceroxylon populations in the northern Andes. (Zorrilla‐Azcué et al., 2021, page 6823, “*Quercus humboldtii* genetic structure” 3rd paragraph)

The second main topic in Hooghiemstra et al. (2022) comment has to do with the interpretation of *Quercus humboldtii*'s past demography. In our original paper, we compared three demographic hypotheses to understand the possible responses of *Q. humboldtii* to the environmental fluctuations of the last glacial–interglacial fluctuations. These corresponded to the following scenarios: (i) constant effective population size; (ii) progressive and constant population expansion; and (iii) a bottleneck event followed by a recent population expansion. The ABC analyses showed that scenario 3 was the best model for explaining *Q. humboldtii* demographic changes. However, Hooghiemstra et al. (2022) suggested that according to palaeoecological understanding, a scenario of constant effective population size is more plausible. Although, they propose that while continuity in oak forest populations was predominant, at a shorter geographic scale, there might be periods of fragmentation among populations. Finally, they disagreed with the appropriateness of the other two demographic hypotheses (scenario 2 and 3).

We would like to address the comment on the conclusion Hooghiemstra et al. (2022) have reached that our proposed demographic scenarios in (Zorrilla‐Azcué et al., 2021) are not appropriate by arguing the rationale behind the two that have been criticized (scenario 2 and 3). However, first, we would like to say that we agree with the proposed hypothesis of different fragmentation processes happening at different time and geographic scales. Nevertheless, given the limiting resolution of the used molecular marker, sampling scheme and modeling approach, we were unable to test more detailed scenarios.

Having said that, both scenarios 2 and 3 have in common a recent increase in population effective size and they were proposed based on different pieces of evidence shown by previous analyses. First, the star‐shaped haplotype network suggests evidence of recent demographic expansion. Secondly, an increase in suitable habitat area obtained by ecological niche models from LGM to PD. Finally, an expectation of an increase in available surface area for establishment as the UFL reached higher altitudes as the interglacial conditions replaced glacial conditions.

Modeling historical demography, in this case with DIYABC, can be assessed by two aspects, model selection and parameter estimation. The former is done by selecting the model whose simulated datasets produce summary statistics that are more similar to the observed summary statistics either directly or through a logistic regression (Cornuet et al., 2008). The models that can be tested need to be simple, and therefore, unavoidably unrealistic, they can only aim to test general tendencies (Cabrera & Palsbøll, 2017). We believe that the thinking behind the proposal of our hypotheses is not necessarily opposed to what *Hooghiemstra* et al are describing in, first:

… the total surface of oak forest was most of the time smaller than today …. (Hooghiemstra et al., 2022, page 3, 3rd paragraph)

and then in,

…progressive and constant population expansion. From a paleoecological point of view this typically occurred at the start of each interglacial period during a few millennia of climate warming. (Hooghiemstra et al., 2022, page 3, 4th paragraph)

Finally, in their synthesis of paleoecological work in 2019, Hooghiemstra and Flantua reported a reduction in available surface area in the LMF of around 42% during the LGM, a vegetation belt which has been mentioned as an important part of the distribution area of *Q. humboldtii*. Based on the preceding observations, we do not think it unreasonable to test scenarios with demographic expansion vs constant population size for recent time periods.

The second part of modeling demographic history is the estimation of parameters. Our interpretation of the results was based on the mean parameter estimation from the posterior distribution of the simulated datasets closest to the observed summary statistics. However, our data have limited power to estimate the exact parameters of timing and population sizes. Some of the reasons being, the large range of confidence intervals, the transformation of time in generations to years (Cabrera & Palsbøll, 2017) and the relative low number of loci we had available (Hoban et al., 2013). We acknowledge our limitations in the understanding of the paleoecological state of the art and appreciate the new insights provided by the authors. At the time, we made the interpretation based on the understanding that the expansion of *Polylepis* might imply a displacement of the altitudinal range in which *Quercus humboldtii* is predominantly abundant and might lead to a change in population size.

We agree with the authors in that through collaboration we can get to more plausible hypotheses that can integrate the evidence provided by both (or even more) fields and are excited by the possibility of working together. However, we do not agree with the conclusion that the proposed models are inadequate. Even with the assumption that the lower limit of altitudinal distribution remained at 800 m everywhere and at all times, there is a reduction in the surface area potentially available for *Q. humboldtii* of ~40% (taking 2000 as the UFL) during the LGM, in comparison with current distribution. This, together with the lack of support for the constant population effective size scenario in our analysis, warrants further investigation using a molecular marker and sampling scheme that can allow us to have higher resolution and more detailed demographic scenarios.

## AUTHOR CONTRIBUTIONS

**Sofia Zorrilla‐Azcué:** Conceptualization (equal); project administration (equal); writing – original draft (equal); writing – review and editing (equal). **Antonio González‐Rodríguez:** Conceptualization (equal); writing – original draft (equal); writing – review and editing (equal). **Ken Oyama:** Conceptualization (equal); writing – original draft (equal); writing – review and editing (equal). **Mailyn A. González:** Conceptualization (equal); writing – original draft (equal); writing – review and editing (equal). **Hernando Rodríguez‐Correa:** Conceptualization (equal); supervision (equal); writing – original draft (equal); writing – review and editing (equal).

## CONFLICT OF INTEREST

The authors have no competing interests to declare.

## Data Availability

All genetic and geographic data from the original paper (Zorrilla‐Azcué et al., 2021) are available in Dryad (https://doi.org/10.5061/dryad.08kprr528).
